# Variation in the Degree of Pectin Methylesterification during the Development of *Baccharis dracunculifolia* Kidney-Shaped Gall

**DOI:** 10.1371/journal.pone.0094588

**Published:** 2014-04-18

**Authors:** Denis Coelho de Oliveira, Thiago Alves Magalhães, Bruno Garcia Ferreira, Cristiane Trindade Teixeira, Anete Teixeira Formiga, Geraldo Wilson Fernandes, Rosy Mary dos Santos Isaias

**Affiliations:** 1 Universidade Federal de Uberlândia (UFU), Instituto de Biologia (INBIO), Campus Umuarama, Uberlândia, Minas Gerais, Brasil; 2 Universidade Federal de Minas Gerais (UFMG), Instituto de Ciências Biológicas, Departamento de Botânica, Belo Horizonte, Minas Gerais, Brasil; 3 Universidade Federal de Minas Gerais (UFMG), Instituto de Ciências Biológicas, Departamento de Biologia Geral, Belo Horizonte, Minas Gerais, Brasil; Universidade Federal de Vicosa, Brazil

## Abstract

Insect galls may be study models to test the distribution of pectins and arabinogalactan-proteins (AGPs) and their related functions during plant cell cycles. These molecules are herein histochemically and immunocitochemically investigated in the kidney-shaped gall induced by *Baccharopelma dracunculifoliae* (Psyllidae) on leaves of *Baccharis dracunculifolia* DC. (Asteraceae) on developmental basis. The homogalacturonans (HGAs) (labeled by JIM5) and the arabinans (labeled by LM6) were detected either in non-galled leaves or in young galls, and indicated stiffening of epidermal cell walls, which is an important step for cell redifferentiation. The labeling of HGAs by JIM7 changed from young to senescent stage, with an increase in the rigidity of cell walls, which is important for the acquaintance of the final gall shape and for the mechanical opening of the gall. The variation on the degree of HGAs during gall development indicated differential PMEs activity during gall development. The epitopes recognized by LM2 (AGP glycan) and LM5 (1–4-β-D-galactans) had poor alterations from non-galled leaves towards gall maturation and senescence. Moreover, the dynamics of pectin and AGPs on two comparable mature kidney-shaped galls on *B. dracunculifolia* and on *B. reticularia* revealed specific peculiarities. Our results indicate that similar gall morphotypes in cogeneric host species may present distinct cell responses in the subcelular level, and also corroborate the functions proposed in literature for HGAs.

## Introduction

The biotic stress induced by the galling herbivores on plant tissues [1], [2], [3], [4], [5] lead to cell redifferentiation [6], which confers them a high adaptive value. Galls function as real plant organs [7], whose cells and tissues have specific structural features [2], and guarantee nourishment, shelter, and protection for the gall inducer and its progeny [8]. As a consequence of the specificity of host plant-galling herbivore systems, unique morphotypes [9] are generated. Their morphogenesis involves subcellular responses in the level of organelles [5], and cell walls, which have been poorly explored by imunocytochemical techniques. These techniques, when applied to identify cell wall compounds, have been setting lights on the knlowledge about the dynamics of cell walls during developmental processes.

Cell wall compounds, the microfibrils of cellulose [10], hemicelluloses, and specially pectins [11], are involved in the process of cell remodeling. Pectins are one of the most abundant components of cell wall matrix [12] and middle lamella, and are considered both structurally and functionally the most complex poslysaccharides in plant cell walls [13]. They may regulate plant growth and defense, morphogenesis and maintenance of the structure, intercellular adhesion, signaling, cell wall expansion and porosity. [11], [12], [14], [15],[16],[17],[18] Their presence has been historically detected by histochemical procedures [19], [20], which are not precise methods to reveal the distinctiveness of the class of pectins, and their functionality during plant cell development. Pectins constitute a diverse class of galacturonic acid-rich polysaccharides (GalA), which can be classified into three main domains: the homogalacturonans (HGAs), the rhamnogalacturonan I (RG-I), and the rhamnogalacturonan II (RG-II) [15], [18], [21]. The HGAs are linear homopolymers of (1–4)-α-linked-D-galacturonic acid with 100–200 GalA residues, which present variation in their degree of methyl-esterification, and correspond to approximately 65% of the total cell wall pectins. The RG-I represents 20–35% of the pectic matrix and is an alternate form of HGAs, which has many structurally different side chains attached via C-4 of the backbone rhamnosil, with arabinosyl and galactosyl residues as the predominant components of the chains. The RG-II is structurally more complex, comprising about 10% of the total pectins in cell matrix. The variation in the methyl-esterification of all these components results in divergence of their physical states, which alters the functional properties of plant cell wall, especially during the growth and development of plant tissues [11], [22], which can be addressed in the cell walls of insect galls. The site of the synthesis of pectins is the Golgi lumen, but how their synthesis are initiated remains unclear [13]. Their demethylesterificaton, however, is performed by enzymes, such as the pectin methylesterases (PMEs), which are encoded by multiple genes classified either as type I or type 2, depending on the presence of a PME inhibitor (type I) or a signal pepitide for secretion (type II) [23]. For cotton fibres, for instance, the two most expressed genes were correlated with the total amount of enzyme activity and de-esterification of pectins at late stages of development. This correlation was observed either biochemically or immunocitochemically [24].

The immunocitochemical labeling of HGAs and RGs with monoclonal antibodies (MAbs) in the cells of insect-induced plant galls may constitute an elegant tool to demonstrate the relationship between pectin distribution and its biological functions at the level of cells and tissues as proposed by Verhertbruggen et al. [25] for plant organs, in general. Also, this kind of analysis can be considered usefull for understanding the patterns of PMEs activity.

Together with the HGAs and RGs, structural and highly soluble glycosylated proteins, the arabinogalactan proteins (AGPs), are associated with the cell wall or the plasmalemma [26]. The AGPs are a class of the hydroxyl-proline-rich glycoprotein family that have mucilaginous appearance and are also involved in cell adhesion [27], plant development, growth, nutrition, cell proliferation [28], [29], and prevention of programmed cell death [30]. They form a gel plug in sites of cell injury constituting a physical barrier to cell invasion [31], specifically by the glycosylation of (1→4)-β-galactans and (1→5)-α-arabinans [18].

The galls on *Baccharis dracunculifolia*, as a model of study of subcellular responses to external stimuli, is evaluated by the comparison of the current results with those presented for *B. reticularia* [32]. On *B. reticularia*, the pectin distribution was studied in three distinct gall morphotypes only in mature phase, while herein we analyse a single and similar morphotype on developmental basis. Current study is based on the premise that pectins are important in the determination of the new shape and functionality of plant galls, which develop through highly specific and repetitive cell cycles [33], [34], and present well-marked developmental phases [2]. The status of the host leaves free of the galling stimuli is herein evaluated as well. If cell wall composition influences on the structure and functionality of the tissues, the kidney-shaped gall morphotype on both *Baccharis* species should present similar immunolabeling for pectins and associated proteins. Our objective is to evaluate histochemically and immunocitochemically the presence and distribution of pectins and AGPs during gall development, checking their association with the functional aspects of gall tissue layers.

## Materials and Methods

### 2.1. Sampling

**Figure 1:**
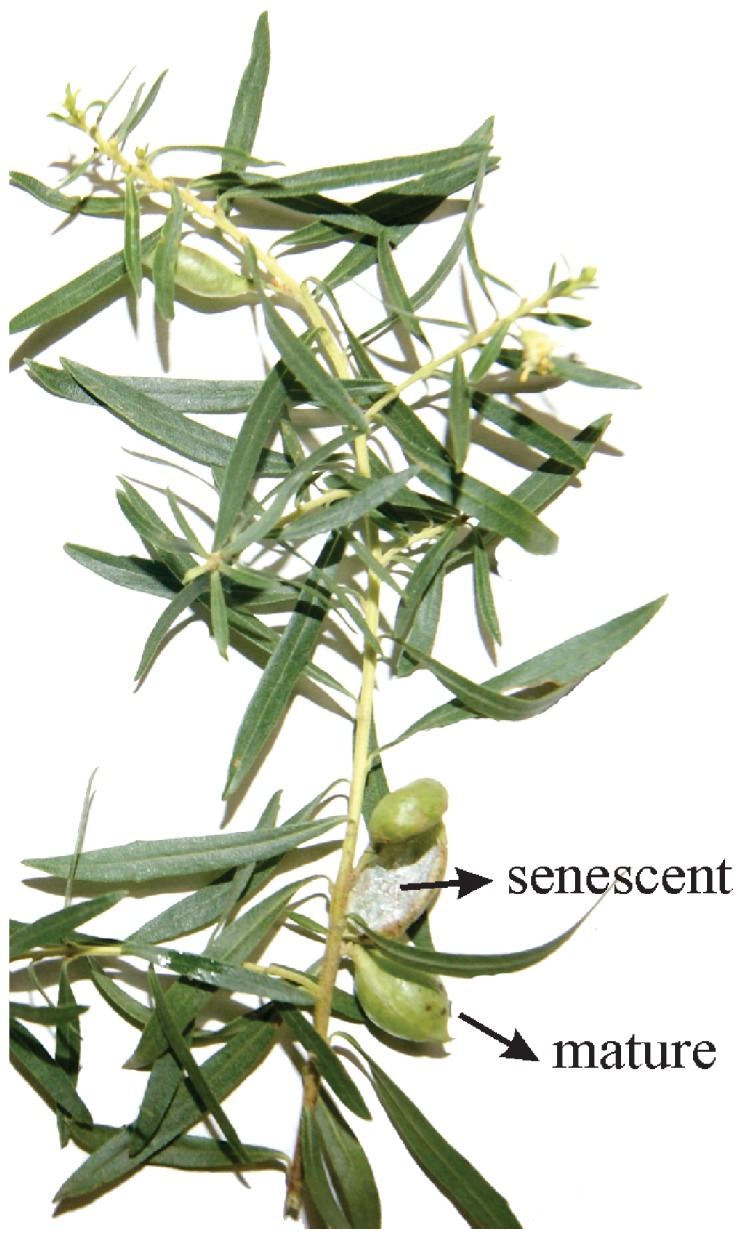
Shoot of *Baccharis dracunculifolia* (Asteraceae) with non-galled leaves and galls of *Baccharopelma dracunculifoliae* (Psyllidae).

The samples consisted of mature non-galled leaves of *Baccharis dracunculifolia* (Asteraceae), and galls induced by *Baccharopelma dracunculifoliae* (Psyllidae) on three developmental stages: young (completely folded leaf), mature (completely folded and swelled leaf with the gall inducer alive) and senescent (open gall, without the gall inducer) (Fig. 1). Collections took place at Serra do Cipó Minas Gerais, Brazil in 2010 and 2011, inside the Private Reserve of the National Patrimony, whose authority responsible is Dr. Geraldo Wilson Fernandes. The samples were collected near state highway (MG10) inside the following coordinates: 19°17′57″S/43°35′58″W.

### 2.2. Histochemistry

Two sets of samples were used for the histochemical detection of pectins. The first set was freehand sectioned and stained with a saturated solution of ruthenium red [35] for unesterified pectins [19] for 15 minutes, washed, mounted in water, visualized and photographed through a light microscope (Leica DM500). The second set of samples was dehydrated in ethanol, embedded in historesin, sectioned in a rotatory microtome, stained in 0.1% coriphosphine for 1 min for detection of esterified pectins [20], and observed under a fluorescence microscope, with UV filter (WU: 330–385 nm), dichroic mirror (400 nm), and barrier filter (420 nm).

### 2.3. Immunocytochemistry

The samples were fixed in 2.5% glutaraldehyde and 2% formaldehyde in 0.1 M phosphate buffer (PBS), pH 7.2 [36], dehydrated in ethanol series, and embedded with historesin Leica®. The sections (5 µm) were obtained in a rotary microtome (Leica BIOCUT® 2035), and incubated with the monoclonal antibodies JIM5, JIM7, LM2, LM5, and LM6 (Centre for Plant Sciences, University of Leeds, UK) (Table 1). The slides were placed in a solution of milk/PBS to prevent crosslinking, incubated with primary antibody (1∶10 diluted in milk/PBS) for 1–2 h, washed in PBS, and subsequently incubated with the secondary antibody FITC Goat anti-rat (Sigma) in milk/PBS for 1–2 h, and washed. The slides were mounted with glycerin and kept in the dark. For negative control, the primary antibody was suppressed. All tests were repeated three times to confirm the results. The images were obtained using the Zeiss 510 META Confocal Microscope, with the excitation Argon laser wavelength of 488 nm, and emission of 505–530 nm.

**Table 1:** Recognition of the monoclonal antibodies to the different epitopes.

Monoclonal antibodies	Epitopes	References
**JIM5**	HGA partially methyl-esterified up to 40%	Clausen et al. [34]
**JIM7**	HGA (15–80% methyl-esterified)	Clausen et al. [34]
**LM2**	AGP glycan	Yates et al.[35], Smallwood et al. [36]
**LM5**	(1→4) β-D-galactan in RG-I	Jones et al. [13]
**LM6**	(1→5) α-L-arabinans in RG-I	Willats et al. [37]

## Results

### 3.1. Leaf and gall anatomy: general features

**Figure 2:**
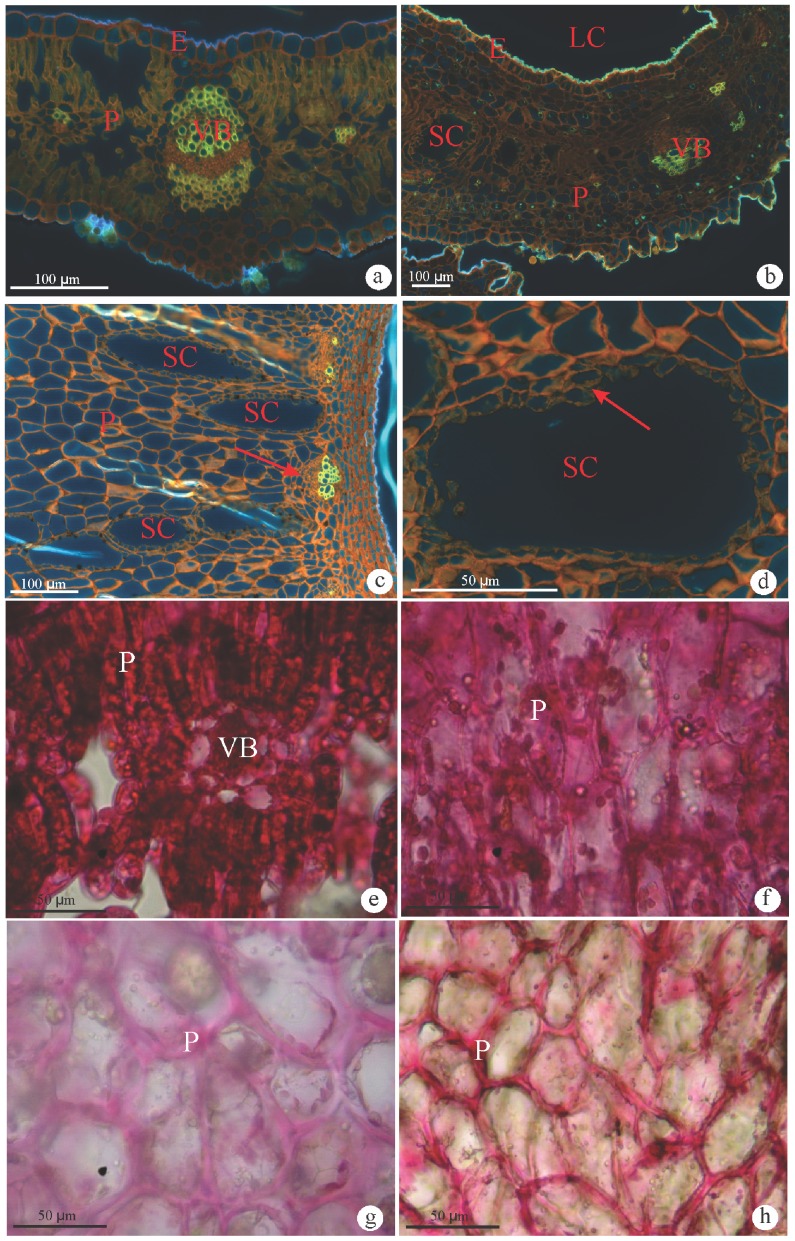
Histochemistry of pectins on transverse sections of non-galled leaves of *Baccharis dracunculifolia* and on galls induced by *Baccharopelma dracunculifoliae*. (a–d) Coriphosphine. (e–h) Ruthenium red. (a) Non-galled leaf. Positive reaction for pectins in cell walls of epidermis, collenchyma, and phloem cells. (b) Young gall. Positive reaction for pectins in epidermis, parenchyma and phloem cells. (c) Mature gall. Intense reaction in the cells near the larval chamber and in phloem cells (arrows). (d) Weak staining at the epithelium (arrow) of a secretory cavity. (e) Non-galled leaf. Intense staining in all tissues. (f) Young gall. Moderate staining. (g) Mature gall. Weak staining. (h) Senescent gall. Moderate staining. E = epidermis; LC = larval chamber; P = parenchyma; SC = secretory cavities; VB = vascular bundles.

The leaves of *Baccharis dracunculifolia* are simple, lanceolate with straight margins (Fig. 1). The epidermis is uniseriate, and the mesophyll is isobilateral with secretory ducts (Fig. 2a). *Baccharopelma dracunculifoliae* induces the folding of the leaf along the midrib with the overlapping of the edges. The resultant gall is green, glabrous, and kidney-shaped at maturity (Fig. 1). The epidermis is uniseriate (Figure 2b) in all developmental stages. Young galls have homogeneous and hyperplasic parenchyma (Fig. 2b). At maturity and senescence, these parenchyma layers are hypertrophied, interspersed with conspicuous vascular bundles, and numerous hypertrophied secretory ducts (Fig. 2c–d).

### 3.2. Hystochemistry

The non-galled leaves present positive and continuous reaction for coriphosphine and ruthenium red in all primary cell walls (Fig. 2a, e). Coriphosphine stained pectins in the cell walls of epidermis and parenchyma of the young (Fig. 2b), mature, and senescent galls (Fig. 2c). The reaction with coriphosphine was comparatively weaker in the cell walls of the epithelium of the secretory ducts (Fig. 2d) than in the other cell tissues. The ruthenium red stained with a lower intensity all primary cell walls in the young, mature and senescent galls (Fig. 2f–h) than in the non-galled tissues.

### 3.3. Immunocytochemistry

**Figure 3:**
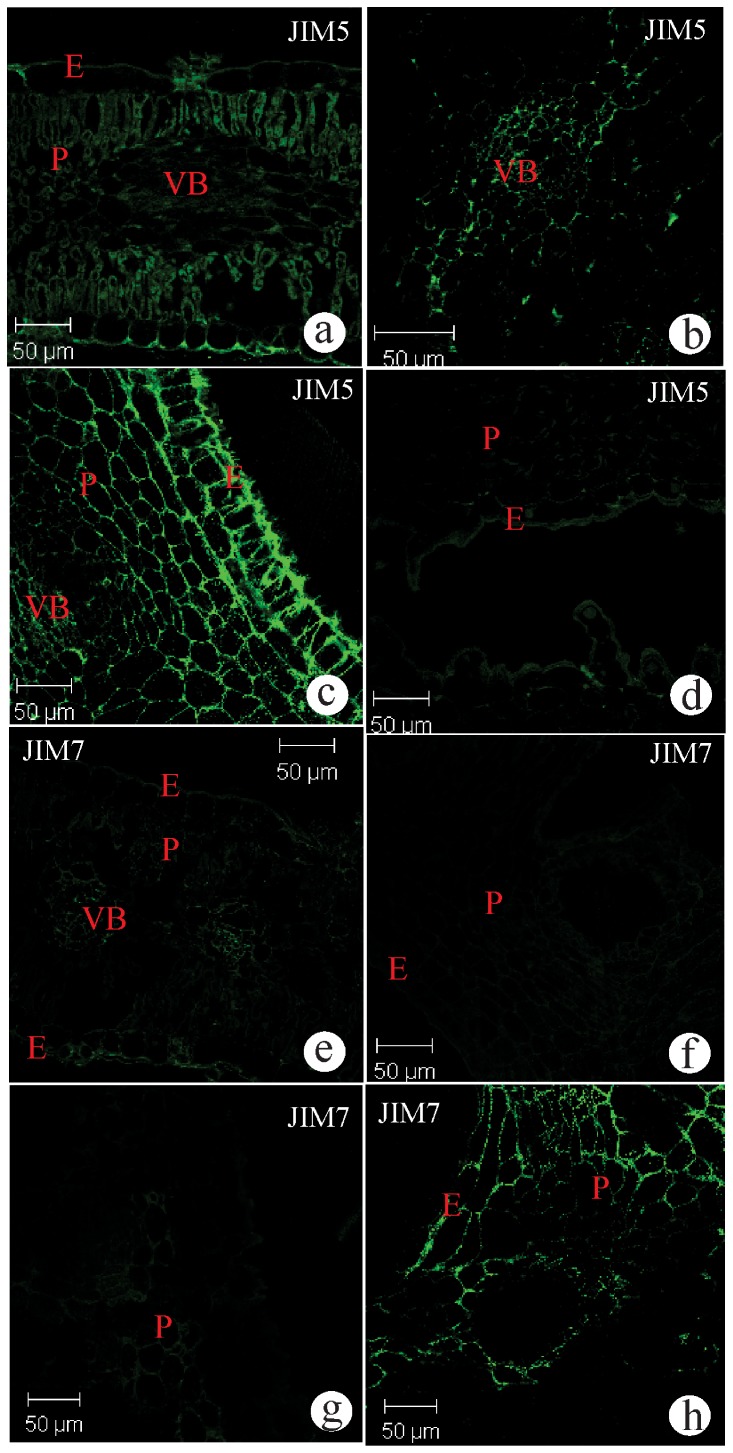
Transverse sections of non-galled leaves of *Baccharis dracunculifolia* and galls induced by *Baccharopelma dracunculifoliae* immunolabeled with the monoclonal antibodies JIM5 amd JIM7. (a–d) JIM5. (a) Non-galled leaf. Moderate labeling in cell walls of epidermis and vascular bundles, and intense labeling in cell walls of parenchyma. (b) Young galls. (b) Detail of the labeling in a vascular bundle. (c) Mature gall. Intense labeling in cell walls of epidermis and parenchyma. (d) Senescent gall. Weak labeling in the cell walls of epidermis and parenchyma. (e–h) JIM7. (e) Non-galled leaf. Moderate labeling in the cell walls of epidermis, parenchyma and vascular system. (f) Young gall and (g) Mature galls. Weak labeling in cell walls of epidermis and parenchyma. (h) Senescent gall. Intense labeling in cell walls of the epidermis and parenchyma. E = epidermis; LC = larval chamber; P = parenchyma; VB = vascular bundles.

The pectins and AGPs epitopes were detected both in non-galled and galled samples, in all developmental stages, but varied in spatial (tissue layers) and temporal (gall stages) distribution (Table 2). In non-galled tissues, the low methyl-esterified epitopes of HGA were intensely bound by JIM5 in the periclinal and anticlinal cell walls of the mesophyll parenchyma, while in the epidermis, the signal was continuous but just in the outer periclinal cell walls (Fig. 3a). In the vascular bundles, the signal for the low methyl-esterified epitopes of HGA was moderate and restricted to the phloem cell walls (Fig. 3a). In young galls, JIM5 reactive cell walls were continuously widespread from the inner epidermis to the adjacent mesophyll parenchyma, but presented unreactive sites towards the outermost cell layers (Fig. 3b). The binding for JIM5 became intense and continuous in the epidermis and parenchyma of mature galls (Fig. 3c). In senescent galls, there was no binding for the JIM5 antibody in any of the tissues (Fig. 3d). The high methyl-esterified HGA epitopes recognized by JIM7 antibody were weakly and discontinuously distributed in the epidermis, parenchyma and phloem cell walls of non-galled tissues (Fig. 3e). In young and mature galls, the JIM7 antibody weakly bound to the high methyl-esterified HGA epitope in the cell walls of epidermis and parenchyma (Fig. 3f–g), while in senescent galls, it is widespread in the cell walls of the parenchyma with some discontinuous areas adjacent to the secretory ducts (Fig. 3h).

**Table 2:** Binding of the antibodies to the pectin epitopes in the non-galled leaves and galls (young, mature and senescent stages) of *Baccharopelma dracunculifoliae* on *Baccharis dracunculifolia*.

Monoclonal antibodies	Tissues	Epidermis	Parenchyma	Vascular system
	Non-galled leaves	++	+++	++
**JIM5**	Young gall	+	+	+
	Mature gall	+++	+++	+++
	Senescent gall	−	−	−
**JIM7**	Non-galled leaves	+	+	+
	Young gall	+	+	−
	Mature gall	+	+	−
	Senescent gall	+++	+++	−
**LM2**	Non-galled leaves	+	++	+
	Young gall	+	+	+
	Mature gall	++	+	+
	Senescent gall	−	−	
**LM5**	Non-galled leaves	+	+	+
	Young gall	+	+	−
	Mature gall	+	+	−
	Senescent gall	+	++	−
**LM6**	Non-galled leaves	+	++	−
	Young gall	++	++	−
	Mature gall	−	+	−
	Senescent gall	−	++	−

The number of signals indicates the intensity of the reaction (+) weak, (++) moderate, and intense (+++).

**Figure 4:**
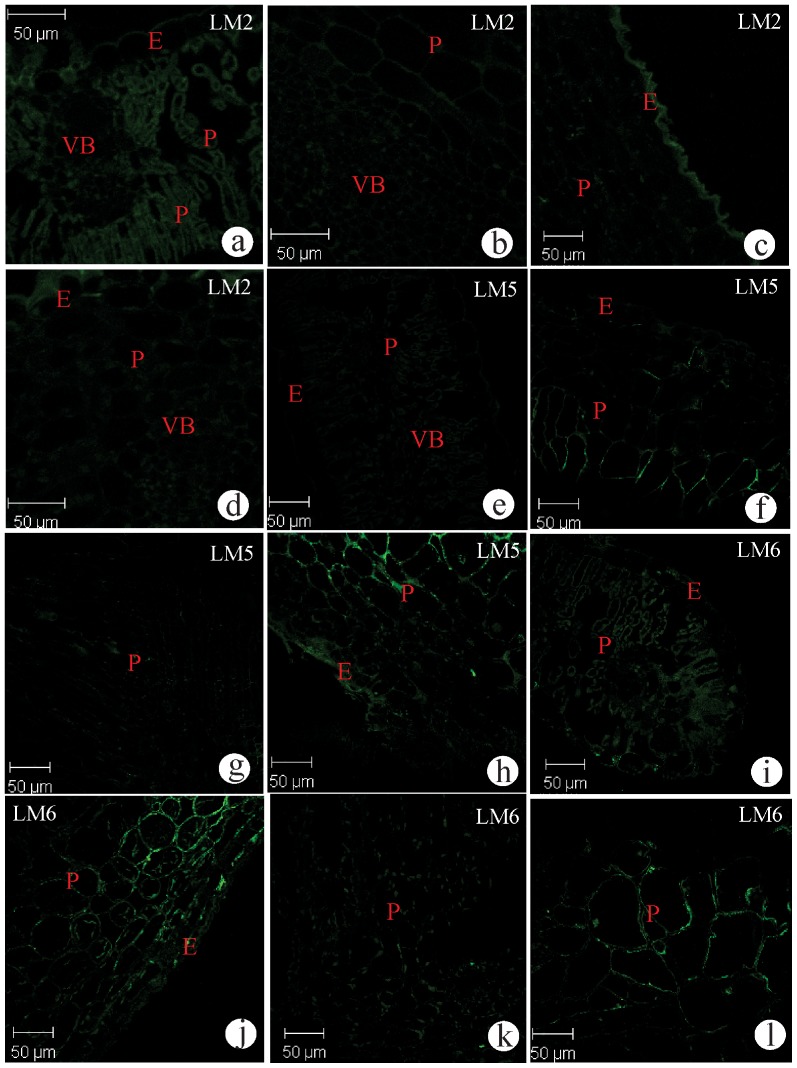
Transverse sections of non-galled leaves of *Baccharis dracunculifolia* and galls induced by *Baccharopelma dracunculifoliae* immunolabeled with the monoclonal antibodies LM2, LM5, and LM6. (a–d) LM2. (a) Non-galled leaf. Weak labeling in the cell walls of epidermis and vascular system, and moderate labeling in the cell walls of parenchyma. (b) Young galls. Weak labeling in the cell walls of the parenchyma, epidermis and vascular bundles. (c) Mature galls. Weak labeling in the cell walls of parenchyma and vascular bundles, and moderate labeling in the cells walls of the epidermis. (d) Senescent gall. Weak labeling in the cell walls of the vascular bundle. (e–h) LM5. (e) Non-galled leaf. Weak labeling in cell walls of epidermis, parenchyma, and vascular bundle. (f) Young and (g) mature gall. Weakly labeling in the cell walls of epidermis and parenchyma. (h) Senescent gall. Moderate labeling in the cell walls of epidermis and parenchyma. (i–l) LM6. (i) Non-galled leaf. Weak labeling in the cell walls of epidermis, and moderate labeling in the cell walls of the parenchyma. (j) Young, (k) mature, and senescent gall (l). Moderate labeling in the cell walls of epidermis and parenchyma. E = epidermis; LC = larval chamber; P = parenchyma; VB = vascular bundles.

The AGPs epitope was continuously bound by the LM2 antibody in the cell walls of the parenchyma either in mesophyll or vascular bundles (Fig. 4a). In young galls, the signals weakened in all tissues (Fig. 4b), but became moderate and continuous just in the outer periclinal cell walls of the epidermis of the mature galls and weak in the senecent galls (Fig. 4c–d). The (1→4)β-D-galactan epitope recognized by LM5 was weakly bound in the cell walls of the non-galled tissues (Fig. 4e), as well as in the cell walls of the epidermis and parenchyma of the young and mature galls (Fig. 4f–g). The reactivity intensified and got a continuous signal in the parenchyma of the senescent galls (Fig. 4h). The (1→5)α-L-arabinan epitope recognized by LM6 was continuous on the parenchyma of non-galled leaves and young galls, and discontinuous for the epidermis of both samples (Fig. 4i–j). In mature galls, the signal weakened, was discontinuous and restricted to the cell walls of the parenchyma (Fig. 4k). In senescent galls, the signals were somewhat more intense, and also restricted to the cell walls of the mesophyll parenchyma (Fig. 4l). The cell walls of the epidermis and vascular bundles were unreactive to the LM6.

## Discussion

The mature kidney-shaped gall of *B. dracunculifolia* has homogenous and parenchymatic mesophyll, small secretory ducts, and unlignified periciclic fibers associated to the vascular bundles [37]. According to this anatomical description, the parenchymatic cells and the secretory ducts hypertrophy as the galls mature. By the time the stimuli for the maintenance of gall tissues stop, the gall naturally opens, and the gall inducer emerges, indicating the existence of transformations in the status of the cell walls, and their components.

One of the cell wall components, the pectins, was histochemically detected in all tissue layers of non-galled and galled samples by the positive reaction to ruthenium red and coriphosphine (Figure 2). This second reagent has revealed a slight difference of pectin detection with a weaker fluorescence in the cell walls of the secretory ducts (Figure 2d). Ruthenium red has been considered a nonspecific reagent for pectins [18], or specific for unesterified (acidic) pectins [19]. The latter author also proposes that ruthenium red stains more intensely the cell walls where de-esterification has occurred, i. e., it may stain more intensely low methyl-esterified pectins and less intensely the high methyl-esterified pectins. The weak staining with ruthenium red in gall samples (Figure 2g–h) may be indicative of pectins de-methylesterification during gall maturation and senescence. The histochemical test with coriphosphine was used to confront these results. Coriphosphine does not react with cellulose, but has affinity to esterified pectins [20]. So, the reduced fluorescence observed in the epithelium (Figure 2d) indicates the presence of low methyl-esterified pectins in the cell walls. Current immunocytochemical analyses labelling of a wide range either of high or low methyl-esterified pectins in non-galled and gall tissues of *B. dracunculifolia* strongly prove the potential of ruthenium red and coriphosphine for the histochemical detection of overall pectins, but did not corroborate their efficiency for evaluating the degree of pectin esterification.

The JIM5 and JIM7 antibodies detected variations on the distribution of pectins in the cell walls of the three plant tissue systems during the development of the kidney-shaped gall of *B. dracunculifolia* (Figure 3, Table 2). The variations in the topography and chemical nature of the pectins should be related to the determination of gall shapes, as a network of the domains of the three polysacharides (HGA, RGI and RGII) can have the potential to modulate cell wall structure [38]. This modulation was previously tested in the kidney-shaped gall of *B. reticularia* [32]. Also, the functional aspects of the gall tissue layers, mainly in relation to cell adhesion and expansion, as well as cell wall porosity, corroborated the assumptions of Knox [14] and McCartney et al. [39].

The degree of methyl-esterification of the homogalacturonans (HGAs) herein analyzed changed from the non-galled tissues towards the senescent galls, which should require the action of pectin methyl esterases (PME) [40] or polygalacturonases (PG) [38]. Moreover, the de-methyl-esterification can be either randomic or linear, with two distinct biological responses. The randomic pattern results in the activation of endopolygalacturonases, which degrade the HGAs and then the whole cell wall [23], [40], which does not seem to have occurred in the tissues of *B. dracunculifolia*, for no cell lysis or death were detected, even in senescent galls. During the linear de-methyl-esterification, the galacturonic acid residues on the HGA chain are hypothesized to interact with calcium ions to form pectate gel, thereby causing cell wall stiffening [41]. At gall maturation, the reactiveness to JIM7 and JIM5 in all tissue layers is indicative of the action of the PME in a linear de-methyl-esterification. Consequently, cell walls are remodeled in such a way that the adhesion between neighbor cells decreases, and the cell walls stiffen, a determinant key process for leaf folding. By senescence, when the gall inducer emerges and cell hypertrophy is over, a decrease of PME activity should occur, leading to the accumulation of high methyl-esterified pectins, as detected by JIM7 [figure 3e–h]. The relative increase in the degree of methyl-esterification of HGAs in senescent galls indicates that just after the emergence of the gall inducer, the gall tissues remain alive and metabolically active. This is another strong indication that the activity of the nymph of *B. dracunculifoliae* is necessary for the maintenance of the PME activity, a metabolic pathway to be explored in the biochemical studies of insect-plant interactions.

In general, it can be assumed that the decrease in the degree of methyl-esterification of the HGAs in the parenchymatic cell walls during gall maturation is necessary given that the cells increase their volumes, as previously observed in the somatic embryos of *Arabidopsis thaliana* [42]. This cell hypertrophy, together with tissue hyperplasia are common processes in gall development [2], [9], [43], and strictly dependent on vacuole turgor, rearrangement of cellulose microfibrils [10], and cell wall extensibility. The de-methyl-esterification of the cell walls has been proved to be a subcellular requisite for these plant cell transformations and occurs in a decreasing centrifugal gradient from the inner epidermis around the larval chamber towards the outer parenchymatic cortex of the gall. This gradient fits the cedidogenous field [44], and indicates that the presence of the nymph of *B. dracunculifoliae* governs the reduction in cell wall extensibility and the cessation of growth, from the outer towards the inner portions of the kidney-shaped gall of *B. dracunculifolia*.

The AGPs epitopes presented decreasing signals from non-galled to galled condition in *B. dracunculifolia*, which contradicts the expected relationship between this proteins and cell metabolism. The AGPs are known to be involved in cell proliferation, cell wall formation [30], and in the control of the programmed cell death (PCD) [30], [45]. The active presence of the nymphs of *B. dracunculifoliae* seems not only to activate PME but to block the synthesis and deposition of AGPs in cell walls, whose consequence is the disruption of normal morphogenesis, as postulated by Cassab [31]. The new morphogenetical pathways observed in the kidney-shaped gall on *B. dracunculifolia* seems to be independent of AGPs regulationn, which culminate with their total absence in senescence, the stage when cell cycles end. This proposal is reinforced by the apoptotic cytological features of senescent galls [2], [3], and was previously reported for old leaves of *Nicotiana tabacum* [45] and for coleoptiles of *Zea mays* [47].

The association of (1→4)β-D-galactans with the HGAs should maintain the mechanical estability of cell wall [45], [48], [49] in senescent galls. The occurrence of few LM5-reactive cells in young and mature galls denotes the specific sites of tissue flexibility necessary for the redefinition of the laminar organization towards the marginal folding of the kidney-shaped gall. The galactans epitope has been reported in mature cells of distinct plant structures, such as mature periderm, mainly in the pheloderm of potato tuber (*Solanum tuberosum* L.) [49], and in the pericarp of tomato (*Lycopersicon esculentum*) [50], where the cells are at the end of their cycles, but the whole structure should keep some extensibility.

Both the (1→5)α-L-arabinans and (1→4)β-D-galactans are absent during maturation of galls, but reappear during senescence, which illustrate the dynamics of cell wall components by the end of gall development on the leaves of *B. dracunculifolia*. The co-occurrence of galactans with the arabinans may influence the alignment of the HGAs [51], contribute to the porosity of cell wall, and consequently to the translocation of substances necessary for the signaling of the end of cell cycles. This is a new insight on the relationship between the structure and function of RGI, which was obscure according to Willats [38].

Current results definitely proved the histochemical reagents, ruthenium red and coriphosphine, are not usefull to indicate the degree of pectin methylesterification. The data obtained by immunocitochemical labeling demonstrated that there was an increase in the degree of methylesterification from young to senescent galls. The differences in the distribution of pectins and AGPs along the development of the kidney-shaped gall of *B. dracunculifolia* is related to the elasticity and porosity in young and mature galls, and to the rigidity acquired from maturity towards senescence. The dynamics of the pectin composition of the cell walls in the galls of *B. dracunculifolia* has revealed to be distinct from that of *B. reticularia* [32]. In the first model, the JIM5 antibody bound to the low methyl-esterified HGAs at the cells of the vascular system just in young galls, while in the second, most of the antibodies bound their related epitopes in the differentiated tissues. The differences herein reported led us to the conclusion that even similar gall morphotypes induced in cogeneric host species may respond distinctly to the galling stimuli in the subcellular level. The galls on *B. dracunculifolia* have proved to the elegant models for the study of the dynamics of cell walls during the cell cycles.The cyclic and repetitive transformations of the cells on galls, and in their distinct tissue layers have confirmed the proposed functions of the HGAs.
